# Relationship of psychological and oral health statuses with self-perceived halitosis in a Jordanian population: a cross-sectional study

**DOI:** 10.1186/s12903-015-0078-7

**Published:** 2015-07-31

**Authors:** Firas Q. Alzoubi, Jumana A. Karasneh, Nidal M. Daamseh

**Affiliations:** Division of Otolaryngology, Department of Special Surgery, Faculty of Medicine, Jordan University of Science and Technology, P.O. Box 3030, Irbid, Jordan; Department of Oral Medicine and Oral Surgery, Dental School, Jordan University of Science and Technology, Irbid, Jordan; Private practice, Amman, Jordan

**Keywords:** Halitosis, Self-perception, Organoleptic test, BANA test, Psychopathology

## Abstract

**Background:**

Self-perceived halitosis could be a symptom of a psychosomatic or psychogenic disorder. The aim of this cross-sectional study was to clarify the relationship of self-perceived halitosis with psychological and oral health statuses.

**Methods:**

One hundred participants with a history of halitosis were enrolled from a teaching hospital. They were divided into the self-perceived and suggested groups if they sensed and did not sense the malodor, respectively. Demographic and socioeconomic information, smoking status, and oral hygiene practices were noted. Complete nasal, oral, and periodontal examinations with organoleptic tests (OLTs) and N-benzoyl-DL-arginine-2-naphthylamide (BANA) tests were conducted. The participants also completed the validated Arabic version of the 90-item revised symptom checklist (SCL-90R). Data were compared by analysis of variance, chi-square test, Student’s *t*-test, and multivariate logistic regression.

**Results:**

The self-perceived group had higher OLT scores (p = 0.005) and were significantly younger (p = 0.001) than the suggested group. A significantly higher number of its participants were smokers (p = 0.004). No significant differences were observed in socioeconomic information, oral hygiene practices, oral conditions, and BANA test results. Further, no significant association was noted between self-perceived halitosis and the nine psychological dimensions of SCL-90R.

**Conclusions:**

Halitosis is a multifactorial symptom that requires multidisciplinary management. Self-reporting of the condition is unique entity and trust worthy symptom. It tends to be related to nonoral pathologies and extrinsic causes such as smoking.

## Background

Halitosis is a frustrating symptom and a reason for frequent primary or secondary care visits. It is associated with a spectrum of disorders across multiple medical specialties, so it poses significant therapeutic challenges for dentists and physicians. Halitosis can originate from oral or nonoral sources [1]. In 90 % of the cases, the causative factor is located in the mouth, such as deep carious lesions, periodontal disease, oral infection, pericoronitis, mucosal ulceration, food impaction, reduced salivary flow, and coated tongue [2]. Nonoral causes include paranasal and laryngeal lesions and systemic diseases such as diabetes mellitus [3].

Many oral bacteria produce volatile sulfur compounds (VSCs) [4]. Their presence in dental plaque or tongue coating is colorimetrically demonstrated by their ability to hydrolyze the synthetic trypsin substrate N-benzoyl-DL-arginine-2-naphthylamide (BANA) [5], producing blue pinpoints or patches, in the BANA test, a modern chair-side method. Further, the organoleptic test (OLT) is the gold standard to detect oral malodor [6], despite the introduction of techniques such as sulfide monitoring and gas chromatography. Instrumental sensors are useful for identifying VSCs alone, whereas the OLT can detect and recognize the compounds in complex mixtures. In addition, it is the only method of assessing the degree of social offensiveness of breath odor [7].

Sometimes, patients sense halitosis but the malodor is neither offensive nor noticeable [8]. Self-perception of halitosis may be related to a psychogenic or psychosomatic disorder [9]. It may indicate depression or obsessive-compulsive behavior, necessitating psychiatric care [10]. Anxiety itself increases the oral levels of VSCs [11], so many professionals do not consider self-reporting of halitosis reliable.

The aim of this cross-sectional study was to clarify the relationship of self-perceived halitosis with psychological and oral health statuses.

## Methods

### Ethical statement

The study was independently reviewed and approved by the Institutional Research Board (IRB) of Jordan University of Science and Technology (JUST). The purpose and methodology of the study, including possible future publication of clinical datasets, were fully explained to all participants and their written consent was obtained before interviews and examinations, complying with the tenets of the revised Declaration of Helsinki.

### Subjects

A single clinician recruited 125 outpatients with a history of halitosis from King Abdullah University Hospital between January and April 2013. These individuals had visited the hospital for nonmedical reasons or problems not acutely related to otolaryngological and oral diseases. They were enrolled if they sensed halitosis (self-perceived group) or were advised about the malodor by a dentist, family member, or friend (suggested group). Those with acute infection, nasal obstruction, history of malignancy, diabetes mellitus, immunosuppression, any systemic condition affecting dental or periodontal disease, pregnancy, and edentulism were excluded.

### Data collection

Demographic and socioeconomic information and smoking status were recorded. Oral hygiene practices were assessed through questions on the frequency of tooth brushing, flossing, miswak (chewing stick) use, and mouth rinse use.

A nasal examination was conducted to exclude nasal masses, septal perforation, and excessive crustation. An oral examination was also performed to detect ulcers, sinus tracks, signs of reduced salivary flow, and tongue coating. Four clinical variables were recorded: the decayed, missing, or filled teeth (DMFT) index, plaque index (PI), gingival index (GI) [12], and clinical attachment loss (CAL), which was measured at six sites around each tooth and averaged (Table 1). The periodontal examination was repeated in 10 participants within 7 days to test intraexaminer reliability: the result showed 97 % agreement between the examinations.

**Table 1:** Diagnostic test scores and definitions in this study

Tongue coating	0: Not apparent
	1: <1/3 of the dorsum coated
	2: 1/3 to 2/3 of the dorsum coated
	3: >2/3 of the dorsum coated
OLT	0: No odor present
	1: Slight malodor (barely noticeable)
	2: Moderate malodor
	3: Offensive malodor (strongly noticeable)
BANA test	0: negative (no blue color)
	1: weakly positive (faint blue color; corresponding to 10^4^–10^5^ colony-forming units)
	2: strongly positive (definite blue color; corresponding to ≥10^6^ colony-forming units)
Chronic periodontitis	Presence of four or more teeth with at least one site having PPD ≥ 4 mm or CAL ≥ 3 mm.
Severity of periodontitis	Percentage of sites affected by periodontal disease (the number of involved sites divided by the total number of sites measured)

*OLT* organoleptic test, *BANA* N-benzoyl-DL-arginine-2-naphthylamide, *PPD* periodontal pocket depth, *CAL* clinical attachment loss

The participants were instructed to avoid drinking, eating, smoking, chewing gum, and mouth rinsing 2 h before the OLT and BANA test. In the OLT, each participant closed the mouth, did not swallow for 60 s, and then exhaled gently through a 10-cm-long tube. The severity of malodor was immediately recorded on a four-point scale (Table 1). The BANA test [Hexagon International (GB) Ltd., Berkhamsted, UK] was performed and interpreted according to the manufacturer’s instructions. Another examiner, who was blinded to the original readings, checked the presence and intensity of colors on the test strips to determine reliability. Agreement between the readings was 98 %.

The 90-item revised symptom checklist (SCL-90R) was used to assess psychological status. It includes three global indices with nine psychological dimensions relevant to general psychiatric distress [13]. The participants were asked to complete the validated Arabic version of the test while waiting [14]. They rated the extent to which each item was manifested during the preceding week using a five-point scale.

### Statistical analysis

SPSS version 17 software (SPSS, Inc., Chicago, IL) was used for data processing and analysis. Categorical variables are presented as number of patients (%) and continuous variables are shown as means and standard deviation. Analysis of variance and the chi-square test were used to determine intergroup differences. SCL-90R data were analyzed by two-tailed Student’s *t*-test at the 5 % significance level. Multivariate logistic regression was used to identify sociodemographic risk factors of psychological symptoms; adjusted odds ratios and 95 % confidence intervals were calculated.

## Results

Of the 125 recruited outpatients, 12 individuals were excluded because of upper respiratory tract, oral, or systemic diseases. Further, 13 participants did not adhere to the study protocol, complete the questionnaire, or have the full examination. Therefore, data of 100 participants (41 men and 59 women) were analyzed.

The mean age of the self-perceived group was significantly lower than that of the suggested group (47 years vs. 55.6 years; *p* = 0.001). Most of the participants were unemployed and fewer than half had a university degree. Further, 67 participants brushed their teeth daily, but the other oral hygiene methods were not commonly used. The self-perceived group had a significantly higher number of smokers (*p* = 0.004; Table 2).

**Table 2:** Intergroup comparison of demographic and socioeconomic data, smoking status, and oral hygiene practices

Characteristic	Self-perceived group (*n* = 50)	Suggested group (*n* = 50)	*P*-value
Mean age (SD), years	47.0 (12.9)	55.6 (13.4)	0.001*
Male gender (%)	24 (48)	17 (34)	0.155
Occupational status (%)			0.204
Employer/professional	10 (20)	5 (10)	
Worker	9 (18)	6 (12)	
Unemployed	31 (62)	39 (78)	
Low monthly income (<500 JOD^a^)	38 (76)	41 (82)	0.461
Educational level (%)			0.588
Primary	17 (34)	22 (44)	
High school	11 (22)	9 (18)	
University	22 (44)	19 (38)	
Smoking status			
Smoker (%)	21 (42)	8 (16)	0.004*
Quantity (>20 cigarettes/day)	12 (57)	4 (50)	0.470
Duration (>10 years)	16 (76)	6 (75)	0.461
Oral hygiene practice (%)			
Brushing (at least once daily)	35 (70)	32 (64)	0.143
Flossing	2 (4)	7 (14)	0.081
Miswak use	8 (16)	8 (16)	1.000
Mouth rinse use	15 (30)	16 (32)	0.829

^a^1 Jordanian Dinar (JOD) equals 1.4 US Dollars

*SD* standard deviation

*statistically significant

The OLT scores showed that most of the participants in the self-perceived group had halitosis (*p* = 0.005; Table 3). When the scores of 0 and 1 were considered negative for halitosis, the sensitivity, specificity, and positive predictive value were 54 %, 67 %, and 88 %, respectively. No significant differences in the presence of coated tongue, fissured tongue, and dental prosthesis or BANA test results were observed between the groups.

**Table 3:** Intergroup comparison of diagnostic test results and oral conditions

Test/condition	Self-perceived group (*n* = 50)	Suggested group (*n* = 50)	*P*-value
OLT score			0.005*
0 or 1	6	12	
2	15	26	
3	29	12	
BANA test score			0.644
0	10	10	
1	24	28	
2	16	12	
Tongue-coating score			0.226
0	7	10	
1	19	17	
2	15	20	
3	9	3	
Fissured tongue	17	16	0.832
Dental prosthesis	22	27	0.403
Periodontitis	22	32	0.067

*OLT* organoleptic test, *BANA* N-benzoyl-DL-arginine-2-naphthylamide

*statistically significant

Regarding the SCL-90R, no significant association was noted between truly self-perceived halitosis (sensed by the participant and detected by the OLT) or delusional halitosis (sensed by the participant but not detected by the OLT) and the nine psychological dimensions (Table 4). Many participants in the self-perceived group had depression or anxiety (aOR = 1.04 and 1.51, respectively), although these results were not significant (*p* = 0.092 and 0.062 respectively).

**Table 4:** Multivariate logistic regression results of the relationship between SCL-90R psychological dimensions and self-perceived halitosis

Dimension	Total self-perceived population (aOR)	*P*-value	OLT positive (aOR)	*P*-value	OLT negative (aOR)	*P*-value
Somatization	1.06	0.715	1.09	0.350	1.04	0.978
Obsessive-compulsive	1.18	0.529	1.20	0.340	1.15	0.987
Interpersonal-sensitivity	0.88	0.560	0.91	0.090	0.86	0.716
Depression	1.02	0.140	1.04	0.092	1.01	0.611
Anxiety	1.48	0.120	1.51	0.062	1.45	0.566
Hostility	0.89	0.562	0.95	0.604	0.83	0.218
Phobic anxiety	0.67	0.792	0.64	0.386	0.652	0.550
Paranoid ideation	0.83	0.248	0.85	0.386	0.816	0.950
Psychotism	1.10	0.268	1.12	0.075	1.09	0.532

*SCL-90R* 90-item revised symptom checklist, *OLT* organoleptic test, *aOR* adjusted odds ratio

## Discussion

Bad breath is a stigma that can affect an individual socially and emotionally. It is an international problem affecting different cultures and societies [15–17]. The global prevalence of halitosis ranges from 15 % to 50 % [18, 19]. In Jordan, its prevalence was recently reported to be 78 %, but the prevalence drops to 36 % if barely noticeable oral malodor (OLT score = 1) is considered negative for halitosis [20].

In this study, we tried to explore the self-perception of halitosis. Because cognitive, emotional, and psychological factors strongly influence its reliability, we included the psychopathological assessment to overcome this limitation. The participants with self-perceived halitosis were almost 9 years younger the other participants. This difference can be explained by the fact that young and middle-aged people tend to be more vigilant and anxious about their health [21].

Hydrogen sulfide, methyl mercaptan, and to a lesser extent, dimethyl sulfide account for 90 % of the VSCs in breath, suggesting that they are responsible for halitosis [22]. Patients with periodontal disease frequently suffer from oral malodor, and a positive correlation has been demonstrated between severity of periodontitis and VSC levels [23]. The OLT suggests halitosis by the intensity of the malodor [10]. On the other hand, the BANA test indirectly indicates oral malodor by detecting red-complex bacteria. The test is negative when halitosis is not caused by these microorganisms or oral conditions, contributing to nearly 85 % of all cases [24]. In this study, the participants with self-perceived halitosis had significantly higher OLT scores but showed no significant differences in oral conditions or BANA test results. These findings suggest that halitosis in people who sense oral malodor might be caused by nonoral factors, such as smoking [25]; smokers constituted 29 % of the study population. The theory also explains the lack of significant differences in oral hygiene practices between the groups.

The participants who sensed halitosis and had a positive OLT finding tended to have depression or anxiety. Emotional status could have a negative impact on body image and the patient may become more sensitive to malodor, causing a multifactorial psychophysiological problem related closely to the psychopathological profile [26]. Two previous studies explored the psychological aspect of halitosis: they showed that a lower severity of halitosis is associated with a stronger psychopathological profile [27, 28]. These studies included patients who attended halitosis clinics, already had the stigma with its psychological burden, and were obviously different from the present sample, who did not seek help. This may explain the lack of significant differences in relation to the SCL-90R findings in our study.

### Limitations of the study

This study has some limitations. First, it was a cross-sectional study of individuals recruited from the outpatient departments of a hospital, so the possibility of selection bias cannot be eliminated. However, we applied strict exclusion criteria and avoided recruiting patients from otolaryngology and oral medicine clinics; the strict criteria can weaken external validity and may not eliminate the bias. Second, the study was designed to include more than 100 participants; the sample size may be insufficient to give a stronger statistical difference. Consequently, larger and more representative community-based studies are required.

## Conclusion

Halitosis is a multifactorial symptom that requires multidisciplinary approach. Self-reporting of the condition is unique entity and reliable symptom. Younger individuals tend to sense oral malodor and seek help. Self-perceived halitosis is mainly related to nonoral pathologies and extrinsic causes such as smoking.
